# Diagnostic accuracy of interleukin-6, interleukin-10 and tumor necrosis factor alpha cytokine levels in patients with mild cognitive impairment: systematic review and meta-analysis

**DOI:** 10.1590/1980-5764-DN-2023-0027

**Published:** 2024-06-24

**Authors:** Alana Mara Inácio de Aquino, Kedma Anne Lima Gomes, Letícia Lorena Melo de Brito, Luciana Domingos de Lima, Eneas Ricardo de Morais Gomes, Suellen Mary Marinho dos Santos Andrade

**Affiliations:** 1 Universidade Federal da Paraíba, Laboratório de Envelhecimento e Neurociências, João Pessoa PB, Brazil.; 2 Universidade Federal da Paraíba, Programa de Graduação em Neurociências e Comportamento, João Pessoa PB, Brazil.; 3 Universidade Federal da Paraíba, Graduação em Fisioterapia, João Pessoa PB, Brazil.; 4 Universidade Federal da Paraíba, Cento de Biotecnologia, Departamento de Biotecnolgia, João Pessoa PB, Brazil.; 5 Universidade Federal da Paraíba, Graduação em Biotecnologia, João Pessoa PB, Brazil.; 6 Universidade Federal da Paraíba, Departamento de Fisioterapia, João Pessoa PB, Brazil.

**Keywords:** Cognitive Dysfunction, Inflammation, Interleukins, Tumor Necrosis Factor-alpha, Disfunção Cognitiva, Inflamação, Interleucinas, Fator de Necrose Tumoral alfa

## Abstract

There is growing evidence suggesting an association between neurodegeneration and inflammation playing a role in the pathogenesis of age-associated diseases, including Alzheimer's disease (AD) and Mild Cognitive Impairment (MCI).

**Objective:**

A systematic review and meta-analysis were performed to verify evidence on the diagnostic accuracy parameters of the inflammatory cytokines interleukin-6 (IL-6), interleukin-10 (IL-10) and tumor necrosis factor alpha (TNF-α).

**Methods:**

A search of Medical Literature Analysis and Retrieval System Online (Medline), Scientific Electronic Library Online (SciELO), Web of Science and Science Direct databases was performed and nine observational studies associated with peripheral inflammatory biomarkers in MCI were identified. Mean (±standard deviation — SD) concentrations of these biomarkers and values of true positives, true negatives, false positives and false negatives for MCI and healthy controls (HC) were extracted from these studies.

**Results:**

Significantly higher levels of IL-10 were observed in subjects in the MCI group and Mini-Mental State Examination (MMSE) scores were lower compared to HC. For the other investigations, no differences were found between the groups. Our meta-analysis for the TNF-α biomarker revealed high heterogeneity between studies in terms of sensitivity and specificity.

**Conclusion:**

These findings do not support the involvement of inflammatory biomarkers for detection of MCI, although significant heterogeneity was observed. More studies are needed to evaluate the role of these cytokines in MCI, as well as in other stages of cognitive decline and all-cause dementias.

## RESUMO

Há evidências crescentes que sugerem uma associação entre a neurodegeneração e a inflamação, desempenhando um papel na patogênese de doenças associadas à idade, incluindo a doença de Alzheimer (DA) e o comprometimento cognitivo leve (CCL).

**Objetivo:**

Uma revisão sistemática e metanálise foram realizadas para verificar evidências relativas aos parâmetros de acurácia diagnóstica das citocinas inflamatórias interleucina-6 (IL-6), interleucina-10 (IL-10) e fator de necrose tumoral (TNF-α).

**Métodos:**

Foi realizada uma busca nas bases de dados Medical Literature Analysis and Retrieval System Online (Medline), Scientific Electronic Library Online (SciELO), Web of Science e Science Direct, e foram identificados nove estudos observacionais associados a biomarcadores inflamatórios periféricos no CCL. As concentrações médias (desvio padrão — ±DP) desses biomarcadores e valores de verdadeiros positivos, verdadeiros negativos, falsos positivos e falsos negativos para CCL e controles saudáveis (CS) foram extraídos desses estudos.

**Resultados:**

Níveis significativamente mais elevados de IL-10 foram observados em indivíduos do grupo CCL e os escores do Miniexame do Estado Mental foram mais baixos em comparação com o CS. Para as demais investigações não foram encontradas diferenças entre os grupos. Nossa metanálise para o biomarcador TNF-α revelou alta heterogeneidade entre os estudos em termos de sensibilidade e especificidade.

**Conclusão:**

Esses achados não apoiam o envolvimento de biomarcadores inflamatórios na detecção do CCL, embora tenha sido observada heterogeneidade significativa. Mais estudos são necessários para avaliar o papel dessas citocinas no CCL, bem como em outros estágios de declínio cognitivo e demências de todas as causas.

## INTRODUCTION

The term mild cognitive impairment (MCI) had its concept refined by Petersen and cols^1^, who defined it for the first time as the initial stage of Alzheimer's disease (AD), predominantly characterized by a decline in memory greater than expected for a specific age, but still not enough for a dementia diagnosis. In 2004, the term was expanded and began to be described not only as a phase of AD, but was divided into four subtypes, thus covering other cognitive domains and being associated with other etiologies^2,3^.

Prevalence studies show conversion rates from MCI to AD of around 10 to 15% per year, while in normal individuals the progression to AD is 1 to 2% per year, although an extensive variation in these rates is admitted due to different methodological research strategies, different cognitive assessment instruments, age of the studied population and variability of operational diagnostic criteria^3,4^.

Given the discrepancies found, studies have sought to identify, with greater precision, useful biological biomarkers that can be correlated to mechanisms that may be involved in the development and progression of the severity of cognitive impairment^5^. Increasing evidence has suggested that high levels of cytokines can activate microglia and astrocytes, that become the main effectors of neuroinflammatory signals, causing neuronal metabolic disorders and excitotoxicity, which contribute to neuronal dysfunction and cognitive deterioration^6^.

Thus, believing that inflammatory processes are part of the brain pathology of AD, studies show cytokines such as interleukin (IL)-6^7–10^ as a marker of systemic inflammation that has been associated with cognitive decline, changes in brain morphology and increased risk of dementia^11,12^. Another potentially cited biomarker is tumor necrosis factor alpha (TNF-α), an important mediator of systemic inflammation and activator of the central innate system. Its high levels in the blood or cerebrospinal fluid (CSF) were observed in patients diagnosed with AD^13,14^, being commonly associated with neuronal dysfunction and death^15^.

On the other hand, research discusses interleukins that can modulate inflammation, such as IL-10, where high levels of this anti-inflammatory cytokine can act to inhibit the production of other cytokines^16^. However, these findings are still confusing and unclear, as well as limited.

In this scenario, the assessment of inflammatory markers in individuals at risk of dementia may reflect a more robust diagnostic measure and the conduct of this research aimed to seek evidence of the diagnostic accuracy parameters of cytokines (IL-6, IL-10 and TNF-α) to resolve doubts and complement the detection of MCI, thus providing support in clinical decision making. We also sought to highlight possible gaps relevant to this topic present in the scientific literature that may highlight the need for new, more careful studies.

## METHODS

### Study design

This is a systematic review with meta-analysis of observational studies. The protocol for this study was designed in accordance with the Preferred Reporting Items for Systematic Reviews and Meta-Analyses checklist (PRISMA)^17^ of diagnostic test accuracy studies and Cochrane Collaboration recommendations. In addition, the study was registered with the International Prospective Register of Ongoing Systematic Reviews (PROSPERO), under registration number ID: CRD42021254894.

### Criteria for selection of studies

This review focused on observational studies in which serum levels of the markers of interest, IL-6, IL-10 and TNF-α, in plasma or serum of patients with MCI were compared. For the meta-analysis, those studies that presented values of true positives (TP), true negatives (TN), false positives (FP) and false negatives (FN) were considered, in addition to informing the blinding of participants and professionals involved in the diagnosis and performance of the blood tests.

### Types of participants

Studies were selected that included participants of both sexes; aged ≥ 50 years; diagnosed with MCI according to the criteria of Petersen^18^, and without a diagnosis of dementia according to the Diagnostic and Statistical Manual–IV (DSM-IV) and the National Institute of Neurology and Communication Disorder and Stroke -The Alzheimer's disease and Related Disorders Association Criteria (NINCDS-ADRDA)^19^.

### Eligibility criteria

Studies were included in this systematic review if they met the following eligibility criteria:

- being associated with inflammatory biomarkers (IL-6, IL-10, TNF-α) as predictors in the diagnosis of MCI;
- including healthy controls (HC) and MCI patients as participants;
- having clearly described inclusion and exclusion criteria;
- describing diagnostic criteria and neuropsychological tests for assessing cognitive function. No restrictions were used for MCI subtypes.

Exclusion criteria included:

- articles that were associated with other diseases and disorders, such as vascular dementia, schizophrenia, depression, and others;
- conference abstracts, letters to the editor, opinion pieces or editorials;
- literature or systematic reviews;
- case studies without group-level statistics;
- studies involving animals or works unrelated to the topic addressed.

All longitudinal studies were considered, provided they met the inclusion and quality criteria.

### Search methods for identifying studies

Two researchers (BLLM and LLD) independently performed data collection using the Medical Subject Headings (MeSH) terms : "mild cognitive impairment", "cognitive dysfunction", "inflammation mediators", "inflammation", "interleukins", "tumor necrosis factor alpha" and other terms with a combination of Boolean operators "AND" and "OR" in the following online databases: Medical Analysis and Retrieval System Online — MEDLINE via PubMed; Scientific Electronic Library Online (SciELO); Scopus (Elsevier); Web of Science (now Clarivate Analytics); and Science Direct, with no language restrictions to capture all possible relevant titles. Manual searches were also performed in the reference lists of included studies, related articles, article citations, and in the gray literature. All steps were completed by August 2022.

The selected studies were reassessed by the other authors (GKAL, GERM and ASSMS) to weigh the potentially eligible works and determine whether they actually met the selection criteria.

**Figure 1:**
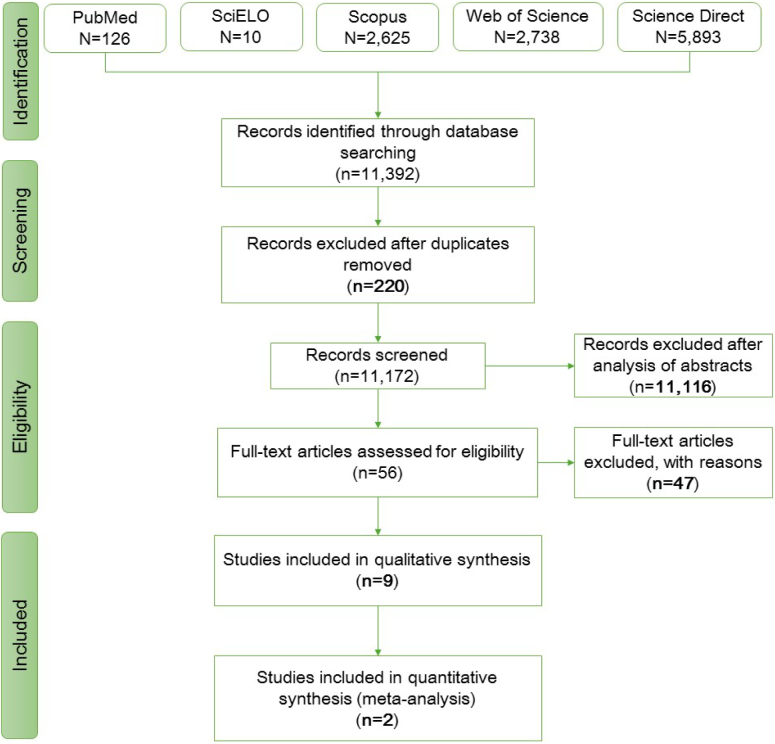
Preferred Reporting Items for Systematic Reviews and Meta-Analyses (PRISMA) flowchart summarizing the study's selection process.

First, there was a reading of the abstracts and a second screening by reading the full text of the other articles. Disagreements were resolved by discussion and consensus with other reviewers, if necessary. All articles were managed in Rayyan Software (Intelligent Systematic Review) which was also used to remove duplicates. Figure 1 shows the flowchart (PRISMA) summarizing the study selection process.

### Data extraction and tabulation

The articles that met the pre-established eligibility criteria were read independently by the researchers (GKAL, GERM and ASSMS) and carefully analyzed to obtain the results. Data were extracted using a spreadsheet with predefined categories, which consisted of general and individual information about each selected study, such as author, year of publication, demographic data of participants (sample size, age, gender, education), mean Mini-Mental State Examination (MMSE) score, values for the diagnostic tests of interest, and whether the sample was collected from serum or plasma. This instrument allowed each reviewer to extract data from the sample individually and facilitated the subsequent analysis of the data obtained. When there was no data or clarity of details, the authors of the studies were contacted for possible clarifications, but only one responded to the researchers’ request for data.

Data from the selected studies were concatenated and examined. The analysis was carried out using the R version 4.2.1 software, freely available at https://www.r-project.org/. The significance level adopted throughout the analysis was 5%.

### Quality assessment and risk of bias

Study quality was assessed using the Revised Tool for the Quality Assessment of Diagnostic Accuracy Studies developed by the University of Bristol, known as QUADAS-2 (https://www.bristol.ac.uk/population-health-sciences/projects/quadas/quadas-2/) and recommended by the Cochrane Collaboration. The risk of bias in individual studies included four domains:

- patient selection;
- index test;
- reference standard; and
- flow and time, analyzing the risk of bias and applicability in each category.

The results were incorporated into our sensitivity analysis, where only studies with low to moderate risk of bias were included. Evaluation was carried out using the Review Manager software version 5.3, and the result of the evaluation of each of the articles is presented in the form of a table.

### Data analysis

At the end of the survey and data organization, a descriptive analysis of the study variables per group (MCI or control) was performed using the mean and standard deviation as summary measures. After the descriptive analysis, in order to choose the appropriate analysis methodology, the Shapiro-Wilk normality test was initially applied to test the null hypothesis that the data follow a normal distribution *versus* the alternative hypothesis that the data do not follow a normal distribution normal. Additionally, to test the hypothesis that there was no difference between the case and control groups for the study variables *versus* the alternative hypothesis that there was a difference between the case and control groups for the study variables, Student's *t* test for samples was used independently for variables with approximately normal distribution. For variables that did not follow an approximately normal distribution, the Wilcoxon-Mann-Whitney test was used.

The meta-analysis was performed according to the technique and sample type of each study (that is, by subgroups). Sensitivity, specificity, positive likelihood ratio (PLR) and negative likelihood ratio (NLR) were measured with a 95% confidence interval based on the TP, TN, FP and FN rates that were extracted from the included studies.

Sensitivity, defined as the probability of a test result being positive when the disease exists (true positive rate) was calculated as = TP /(TP + FN). Specificity, defined as the probability of a test result being negative when the disease is not present (true negative rate) was calculated as = TN/(TN + TP). The PLR is the ratio of the probability of a positive test result given the presence of the disease to the probability of a positive test result given the absence of the disease, that is = true positive rate/false positive rate, or expressed as sensitivity/(1-specificity). The NLR is the ratio between the probability of a negative test result given the presence of the disease and the probability of a negative test result given the absence of the disease, that is = false negative rate/true negative rate. Summary receiver operating characteristics (SROC) curves based on TP and FP rates were also constructed whenever possible to describe the relationship between test sensitivity and specificity.

The heterogeneity of the studies was established using the χ^2^ test, with inconsistency values (I^2^) greater than 50% considered moderate heterogeneity, and I^2^ greater than 75% defined as high heterogeneity. Results with I^2^ values greater than 50% were subjected to sensitivity analysis (that is, hypothetical removal of studies).

## RESULTS

### Identification of studies

The search in the informed databases found a total of 11,392 articles, of which 11,172 were identified after removing duplicates. Of these, 11,116 were excluded during the screening phase (reading the title and abstract) as they did not fit the research profile, with 56 records being fully evaluated. Finally, nine studies that met the eligibility criteria established by the researchers were included in the systematic review, and of these, only two could be included in the meta-analysis. The report items involved in the process of identification and selection of studies are detailed in Figure 1.

The included articles comprised a total of 2,436 participants, of which 653 were allocated to the MCI group, while 1,783 were allocated to the control group. The common information of most studies concerns the size and type of sample, age, sex, MMSE scores, serum IL-6, IL-10 and TNF-α values, and is shown in Table 1 ^20–28^.

**Table 1:** Data.

Study	Typ	Sample		Age		% Women		MMSE		IL-6		IL-10		TNF -α	
		MCI	ctrl	MCI	ctrl	MCI	ctrl	MCI	ctrl	MCI	ctrl	MCI	ctrl	MCI	ctrl
Zhao et al.^20^	serum	150	150	70.67	69.85	47.30	41.30	26.55	27.06	1.86	1.72	-	-	-	-
Wennberg et al.^21^	plasma	186	1416	79.70	71.90	40.90	47.30	-	-	3.10	2.50	0.91	0.80	3.30	4.30
Shen et al.^22^	plasma	57	57	68.77	67.77	31.58	31.58	25.82	29.46	-	-	-	-	15.43	14.29
Magalhães et al.^23^	serum	55	42	71.00	68.00	63.54	71.43	28.51	25.80	3.75	3.40	1.08	0.71	4.86	3.76
King et al.^24^	plasma	77	20	76.13	75.90	40.00	25.00	26.47	29.10	2.07	1.70	1.03	0.50	1.70	4.30
Johansson et al.^25^	serum	11	18	72.00	76.00	54.54	44.44	28.00	29.00	0.88	1.04	-	-	-	-
Kim et al.^26^	us	29	28	75.03	72.00	61.50	57.10	23.62	27.41	9.72	4.60	-	-	5.89	4.60
King et al.^27^	plasma	58	20	77.05	75.90	45.76	25.00	26.45	29.10	3.11	1.66	0.95	0.46	2.66	4.26
Dursun et al.^28^	serum	30	32	74.40	72.10	-	-	27.59	28.57	-	-	-	-	-	-

Abbreviation: MCI: mild cognitive impairment; ctrl: control group.

Additionally, we note that the included studies were published between 2012 and 2019, and that four of them used plasma samples, four used serum samples, and one did not specify the type of sample. Seven of the nine studies included participants aged 60 years or older, and two had participants aged 50 years or older^20,21^. Only one study did not specify the male/female ratio, but considering the others, a total of 1,279 men (339 cases and 940 controls) and 1,095 women (284 cases and 811 controls) were included. The study by Wennberg et al.^21^ was the only one that did not use the MMSE as part of the neuropsychological assessment. Eight of the nine studies evaluated serum levels of IL-6, four of IL-10 and six of TNF-α. As a method used to evaluate cytokine levels, six studies used immunoassays^20,21,24,26–28^, two used luminex assays^22,25^ and one used flow cytometry^23^.

### Comparison between groups

**Figure 2:**
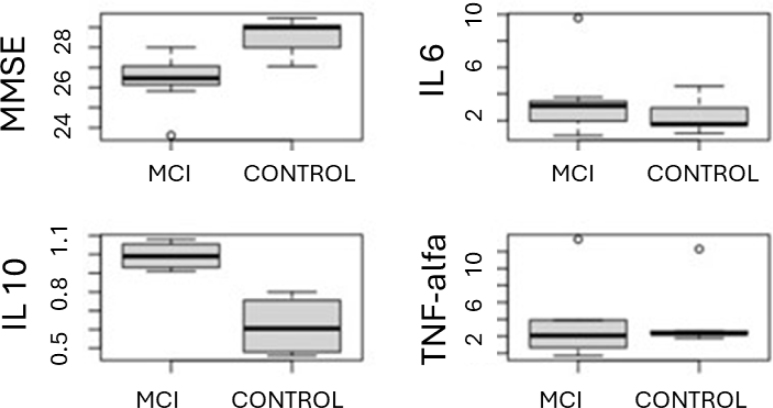
Boxplot of the main study variables by group.

After carrying out the descriptive analyses of the study variables that are available in Table 2, we can observe that there was a significant difference between the MMSE values of the MCI and control groups (p=0.0065), so that the control group presented a higher mean value than the MCI group. There was also a significant difference between the IL-10 values of the MCI and control groups (p=0.0126) so that individuals in the MCI group had a higher mean value than individuals in the control group. We can better observe the results obtained in the comparison between the groups of the main variables of the study in Figure 2.

**Table 2:** Comparison of study variables between groups.

Variable	MCI (n=653)	Control (n=1,783)	p-value
Age	73.86±3.51	72.158±3.25	0.3020*
% Women	48.14±11.06	42.89±16.07	0.4611*
MMSE	26.63±1.51	28.19±1.29	0.0442*,†
IL-6	3.50±2.91	2.37±1.24	0.3735*
IL-10	0.99±0.08	0.62±0.16	0.0126*,†
TNFα	5.64±5.03	5.92±4.11	0.8099^2^

Abbreviations: MMSE, Mini-Mental State Examination; MCI, mild cognitive impairment.

* Notes: Student's *t* test for independent samples;

† significant results; ^‡^ Wilcoxon-Mann-Whitney test.

### Quality assessment

Studies were classified as moderate overall methodological quality according to QUADAS-2. All studies described patient selection methods and those included corresponded to the review question. Overall, the studies adequately reported the index and reference standard tests and how they were conducted and interpreted, but six of the nine studies did not report blinding between those involved in clinical diagnosis and analyses, and only five studies specified the lower limits of detection for the evaluated markers, which entailed implications for the applicability domain.

Those that reported the interval between tests, whether patients received different index or standard trials, and full statistical analyzes performed, were thus judged to have low risk of bias for the flow and time domain. The remaining studies were classified as having an uncertain risk of bias for this domain. The entire summary of the methodological quality assessment can be seen in Supplementary Material Figure S1 (https://www.demneuropsy.com.br/wp-content/uploads/2024/03/ANP-2023.0027-Supplementary-Material-Figure-1.png) and Figure S2 (https://www.demneuropsy.com.br/wp-content/uploads/2024/03/ANP-2023.0027-Supplementary-Material-Figure-2.png).

### Meta-analysis

**Figure 3:**
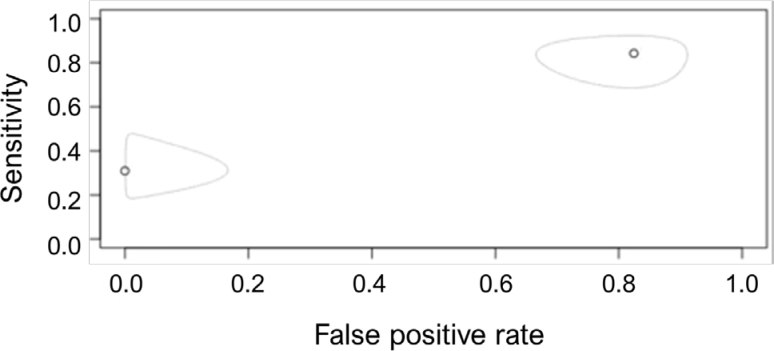
Elliptical roc curve for sensitivity and false positives.

After selecting the studies, a diagnostic meta-analysis was performed for the inflammatory marker TNF-α, as a means of detecting MCI. The studies considered were Shen et al.^22^ and Magalhães et al.^23^. Table 3 describes the sensitivity and specificity values of each study, as well as their respective 95% confidence intervals. We can observe that in the study carried out by Shen et al.^22^, the method had a sensitivity of 0.836 and a specificity of 0.181, that is, we concluded that the method is more likely to be correct in detecting the disease in sick patients than detecting its absence in non-ill patients. In the study carried out by Magalhães et al.^23^, the finding was in the opposite direction, with a sensitivity of 0.321 and specificity of 0.988, which indicates that the method is more effective in detecting the absence of the disease in non-ill patients than the presence of the disease in patients. The results in Table 3 can be seen graphically in Supplementary Material Figure S3 (https://www.demneuropsy.com.br/wp-content/uploads/2024/03/ANP-2023.0027-Supplementary-Material-Figure-3.png) and Figure S4 (https://www.demneuropsy.com.br/wp-content/uploads/2024/03/ANP-2023.0027-Supplementary-Material-Figure-4.png), respectively.

**Table 3:** Sensitivity and specificity of the studies in the meta-analysis.

Study	Sensitivity	Specificity
Shen et al.^22^	0.836 [0.721; 0.910]	0.181 [0.103; 0.299]
Magalhães et al.^23^	0.312 [0.206; 0.443]	0.988 [0.898; 0.999]

### Investigation of heterogeneity and publication bias

To check for heterogeneity between the sensitivity and specificity of the studies, the χ^2^ test was used to test the null hypothesis that the studies have the same sensitivity or specificity *versus* the alternative hypothesis that the studies do not have the same sensitivity or specificity. In both cases, we obtained a p<0.0001, that is, we rejected the null hypotheses that the sensitivity and specificity of the studies are equal. Therefore, based on the sample and with 95% confidence, we conclude that the studies are heterogeneous in terms of sensitivity and specificity. It is important to determine the reason for the variation and understand whether it occurred by chance.

Table 4 ^22,23^ describes the values of the odds ratio of diagnosis or *diagnosis odds ratio* (DOR), the PLR and the NRL from each study, as well as their respective 95% confidence intervals. The PLR ranges from 1 to infinity, and a PLR of 1 indicates that the probability of a positive test result is the same for patients with and without the disease. The NRL ranges from 1 to 0, and the closer the NRL is to 0, the lower the probability of disease in the presence of a negative test result. We can observe that in the study developed by Shen et al.^22^, a PLR=1.021 was obtained, indicating that the test was not very useful due to the fact that 1 belongs to the 95% confidence interval. In that same study, the NRL value was 0.905 and we also observed that 1 belongs to the confidence interval, indicating that the probability of the disease in the negative presence of the test is not small. In the study developed by Magalhães et al.^23^, we observed a PLR value=38.636, indicating that the test has a high probability of being positive in patients. We also have for this study the value of NRL=0.696, indicating a low probability of detecting the disease in non-ill patients.

**Table 4:** Diagnosis odds ratio, positive likelihood ratio and negative likelihood ratio in the meta-analysis.

Study	DOR	PLR	NRL
Shen et al.^22^	1.129 [0.430; 2.961]	1.021 [0.865; 1.206]	0.905 [0.407; 2.011]
Magalhãs et al.^23^	38.636 [2.247; 664.439]	26.875 [1.662; 434.454]	0.696 [0.581; 0.832]

Abbreviations: DOR, diagnosis odds ratio; PLR, positive likelihood ratio; NRL, negative likelihood ratio.

To verify whether the heterogeneity of the studies is due to random effects, the Cochran's Q test was used. The p-value found was 0.317, that is, we do not reject the hypothesis that the heterogeneity is in fact due to random factors, which is corroborated by the inconsistency measure (I^2^), which was approximately 0%. We can also propose a ROC "curve", keeping in mind that, in its classic use, the values are continuous, while in the diagnostic meta-analysis, each study corresponds to a point on the graph, thus not forming a proper curve. The elliptical ROC curve can be seen in Figure 3, in which the points indicate the sensitivity values and the false positive rate of each study, while the ellipse indicates the region of confidence for these values. The optimum point on the graph is the upper left corner, where the sensitivity value is close to 1 and the false positive rate is close to 0. Note that the study by Magalhães et al.^23^ obtained a false positive value close to 0, while the study by Shen et al.^22^ presented a false positive rate greater than 0.8.

## DISCUSSION

In this study, we sought to provide evidence linking inflammation to MCI by investigating the diagnostic accuracy of three particular inflammatory markers, including pro-inflammatory IL-6, TNF-α and anti-inflammatory IL-10. However, it was not possible to verify the diagnostic accuracy due to discrepancies between the studies, in addition to the lack of essential data such as information on the rates of PT, TN, FP and NF, which were only reported by two authors.

Thus, the studies that were included in the meta-analysis for the inflammatory marker TNF-α as a way of detecting MCI pointed to the conclusion, based on the sample and with 95% confidence, that they are heterogeneous in terms of sensitivity and specificity of the tests performed. It must be taken into account that this high heterogeneity may have occurred due to the different methodologies chosen by the studies. One of them used the luminex assay, while the other assessed cytokine levels using flow cytometry. There was also no significant difference after descriptive analysis between the MCI and control groups for the TNF-α marker. This result is in line with that presented by Gezen-Ak et al.^29^, where serum TNF-α levels increased significantly only in the early-onset and late-onset AD groups, and, although these levels were higher in the MCI group than in the age-matched control group, they were not statistically significant. On the other hand, previous research observed a significant increase in serum TNF-α levels in patients with AD and also in the MCI group compared to HC^30–32^, which raises caution, as this is one of the main inflammatory cytokines produced by activated astrocytes and microglia and is increased in the affected brain regions of patients with AD^33^.

Regarding IL-10, the study by Rosenberg et al.^34^ reported that the increase in TNF-α levels stimulates the expression of IL-10, which is an anti-inflammatory cytokine capable of inhibiting its synthesis. In this research, IL-10 levels showed a significant difference between the values of the MCI and control groups (p=0.0126) so that individuals in the MCI group had a mean value higher than those in the control group.

A meta-analysis evaluating the association between peripheral IL-6 levels in all-cause dementia found that higher concentrations of this inflammatory marker conferred an increased risk of developing AD. The authors highlight that peripheral inflammation can occur before clinical symptoms are present^35^. In the study in question, no significant differences were observed in IL-6 levels between the MCI and control groups, which also corroborates the data by Bermejo et al.^36^, where significantly elevated levels of IL-6 were found only in the plasma of patients with AD, while similar values were observed for the MCI group and healthy controls. One of the factors that can explain these results is that, in general, increased levels of IL-6 can be associated with several conditions, including aging itself^37^.

To complement what has already been discussed, another important relationship to be observed is the association of the aforementioned biomarkers with the MMSE scores in these patients. Sharma et al.^38^ did not find strong evidence of associations between inflammatory biomarkers and modified MMSE scores. Gezen-Ak et al.^29^ showed that patients with early-onset AD with low MMSE scores have high serum levels of the TNF-α marker. A significant difference between the MMSE values of the MCI and control groups (p=0.0065) was found in this study, so that the control group had a higher mean value than the MCI group. However, further studies need to elucidate which inflammatory mechanisms directly influence the cognition of these individuals.

### Limitations

This meta-analysis has strengths and limitations. Its strengths include a comprehensive research methodology, as well as being the first study to assess the diagnostic accuracy of cytokines IL-6, IL-10, TNF-α as possible early markers of MCI. Furthermore, few studies presented robust analyses in an attempt to detect possible inconsistencies in the evaluated tests and identify the reason for the high variation reported between them. Our study's limitations are inherent to observational studies that may affect the researchers’ ability to infer any causal relationship between inflammatory markers and MCI, as increased inflammatory levels can be both a cause and a consequence. Another limitation is the small number of studies included, providing little important data available, which made some comparisons unfeasible, restricting the exploration of potential sources of heterogeneity, such as comorbidities, level of physical activity, genetic factors, depression, among others that could elucidate the controversial results and better guide clinical practice.

In conclusion, this review found no evidence suggestive of increased peripheral levels of inflammatory markers in MCI patients. To confirm the findings of this meta-analysis, future observational studies including additional information from the participants are needed to provide a more adequate context, in addition to better assessing the differences in cutoff points between the developed analyses, which may have led to the high degree of heterogeneity presented.
